# Simulating Spatiotemporal Dynamics of Sichuan Grassland Net Primary Productivity Using the CASA Model and In Situ Observations

**DOI:** 10.1155/2014/956963

**Published:** 2014-08-27

**Authors:** Chuanjiang Tang, Xinyu Fu, Dong Jiang, Jingying Fu, Xinyue Zhang, Su Zhou

**Affiliations:** ^1^Sichuan Grassland General Work Station, Chengdu 610041, China; ^2^Institute of Geographical Sciences and Natural Resources Research, Chinese Academy of Sciences, Beijing 100101, China

## Abstract

Net primary productivity (NPP) is an important indicator for grassland resource management and sustainable development. In this paper, the NPP of Sichuan grasslands was estimated by the Carnegie-Ames-Stanford Approach (CASA) model. The results were validated with in situ data. The overall precision reached 70%; alpine meadow had the highest precision at greater than 75%, among the three types of grasslands validated. The spatial and temporal variations of Sichuan grasslands were analyzed. The absorbed photosynthetic active radiation (APAR), light use efficiency (*ε*), and NPP of Sichuan grasslands peaked in August, which was a vigorous growth period during 2011. High values of APAR existed in the southwest regions in altitudes from 2000 m to 4000 m. Light use efficiency (*ε*) varied in the different types of grasslands. The Sichuan grassland NPP was mainly distributed in the region of 3000–5000 m altitude. The NPP of alpine meadow accounted for 50% of the total NPP of Sichuan grasslands.

## 1. Introduction

Net primary production (NPP) represents the accumulated organic matter by plants per unit area and time. From an ecological perspective, it measures the rate at which solar energy is stored by plants as organic matter [1]. NPP is influenced by climate, soil, vegetation type, and human activities [2]. For various ecological monitoring activities, NPP is generally regarded as an important factor that provides a comprehensive evaluation of ecosystem status and services, including productivity capability, habitat, and wildlife, and ecological footprint [3, 4].

NPP is not a directly observable ecosystem characteristic, and it is difficult to measure accurately over large areas due to the spatial variability of environmental conditions [5, 6]. A number of NPP models for different ecosystems have been developed. These models are broadly classified into regression-based and process-based. Regression-based models are established by empirically derived relationships between climate values and NPP, such as Miami [7]. Although regression-based models, with the advantages of simplicity and fewer parameter requirements, can be extrapolated for most land ecosystems, uncertainties are also involved when considering heterogeneous vegetation, standard errors of measurements, and novel climatic conditions, which may not be appropriate for the regressions [8, 9]. Process-based models, ranging from simple models based on light use efficiency (LUE) to more mechanistic models based on “soil-vegetation-atmospheric-transfer” (SVAT) schemes, are based on physiological and ecological processes such as photosynthesis, evapotranspiration, respiration, and nutrient cycling [10, 11]. These models have more parameter requirements and complexities; however, they better describe mechanisms and have the potential to estimate NPP more accurately when compared with regression-based models. The models based on LUE are called production efficiency models (PEMs), which use LUE for the conversion of absorbed photosynthetically active radiation (APAR) to biomass [12]. They are widely acceptable to map NPP at different scales as it follows the basic principles of the photosynthesis process and is easily amenable to remote sensing data [13]. The satellite data-driven PEMs, such as CASA [14], TURC [15], and GLO-PEM [16], have been used to analyze the spatiotemporal patterns of NPP over continents and global land surfaces [17–20].

Grasslands are the largest terrestrial ecosystem in China and account for 41.7% of China's total area [21, 22]. Sichuan province, located in southwest China, is one of the country's most important pastoral areas, with 43% of the area covered by grassland, and available natural grassland accounts for 85% of the total grassland area [23]. In total, 78% of Sichuan grassland is distributed in the northwest and southeastern edge of the Tibetan Plateau, where the upper reaches of the Yangtze and Yellow Rivers flow and the ecological environment are vulnerable and sensitive to climate change and human activities. NPP is an indicator of ecosystem health and ecological balance, and its spatiotemporal pattern is significant for scientific management, reasonable planning, and sustainable development. However, there are few studies that have accurately simulated spatiotemporal dynamics of the entire Sichuan grassland net primary productivity due to the large area, complex topography, and limited field survey work of Sichuan grasslands; however, multiple studies have been conducted in small local areas.

The purpose of this study was to simulate the entire Sichuan grassland net primary productivity and then analyze the spatiotemporal dynamics through parameter calibration and verification for a widely accepted model. The CASA (Carnegie-Ames-Stanford Approach) model was adopted in this study. The CASA model is one of the most popular satellite-driven models. It simulates NPP directly instead of separately calculating GPP, thus avoiding a Ra (autotrophic plant respiration) calculation and taking environmental conditions (temperature, rainfall/soil moisture) and vegetation characteristics into consideration [6, 24]. The CASA model was first introduced by Potter et al. (1993) based on Monteith's equation (1972) and was expanded by Field et al. (1995) using a combination of ecological principles, satellite data, and surface data to predict terrestrial NPP on a monthly time step [14, 25, 26]. To date, the model has been implemented to estimate regional and continental patterns of NPP and has been evaluated for various regions [27–30]. In this study, results of the CASA model were validated by in situ data, derived from the grassland resource survey of the Sichuan province in 2011. Spatial and temporal variation in the APAR, LUE, and NPP of Sichuan grasslands was also analyzed.

## 2. Materials and Methods

### 2.1. Study Area

Sichuan province (26°03′~34°19′N, 97°21′~108°31′E) is located in southwest China. The eastern part of the province is mostly within the Sichuan basin, whereas the west consists of numerous mountain ranges forming the easternmost part of the Tibetan Plateau. Various types of grasslands in Sichuan province are distributed in the 270–5500 m altitude region, and 78% of its grasslands are distributed in the northwest area of Sichuan province, with an elevation of 2800 to 4500 m. The three largest types of grasslands by area are alpine meadow (49%), alpine shrub grassland (15%), and mountain shrub-tussock grassland (9%). The mean annual temperature varies from −1.6°C to 3.3°C. The average monthly precipitation is 78.4 mm, and approximately 90% of the precipitation falls in the growing season from April to October. Subalpine meadow is the dominant soil type [31].

### 2.2. Determining Key Parameters of the CASA Model

The CASA model computes NPP as a function of absorbed photosynthetically active radiation (APAR) and light use efficiency (LUE) [14, 26] as follows:

(1) NPP(x,t)=APAR(x,t)×LUE(x,t),

where *x* represents the grid cell and *t* represents the period in which NPP is accumulated, for example, a month. APAR is determined by the fraction of photosynthetically active radiation (FPAR) and the total solar surface radiation (SOL) (MJ·m^−2^) [32] as

(2) APAR(x,t)=SOL(x,t)×FPAR(x,t)×0.5,

where the constant 0.5 represents the ratio of the total solar radiation (with a wavelength range of 0.4–0.7 *μ*m) used by the vegetation [34].

LUE is calculated as the product of maximum light use efficiency and its temperature and moisture stressors [26] as

(3) LUE(x,t)=Tε1(x,t)×Tε2(x,t)×Wε(x,t)×εmax⁡,

where LUE(*x*, *t*) represents the actual light use efficiency, *ε*_max⁡_ is the maximum light use efficiency, and the value for grass (0.604 g/MJ), simulated by Running based on BIOME-BGC model [35], was used here; *T*_*ε*1_(*x*, *t*) and *T*_*ε*2_(*x*, *t*) are temperature scalars and *W*_*ε*_(*x*, *t*) is the moisture stress coefficient. *T*_*ε*1_(*x*, *t*), *T*_*ε*2_(*x*, *t*), and *W*_*ε*_(*x*, *t*) were computed at every location at each time step. *T*_*ε*1_(*x*, *t*) and *T*_*ε*2_(*x*, *t*) are calculated as [26, 32]

(4) Tε1(x)=0.8+0.02×Topt(x)−0.0005×[Topt(x)]2,Tε2(x,t)=1.1814×1{1+exp⁡[0.2×(Topt(x)−10−T(x,t))]}×1{1+exp⁡[0.3×(−Topt(x)−10+T(x,t))]},

where *T*_opt_ is an optimal temperature, defined as the mean temperature in the month of maximum NDVI. *T* is the monthly mean temperature; *T*_*ε*2_(*x*, *t*) = 1, when *T* = *T*_opt_; it decreases to 0.5 when *T* is 10°C above or 13°C below *T*_opt_.

*W*_*ε*_(*x*, *t*) reflects the effect of water condition, and it generally increases when available water increases. Atmospheric vapor pressure deficit reflects air humidity, which affects transpiration and then the LUE [36]. Therefore, there are currently studies using vapor pressure deficit (*D* in kPa) to calculate the moisture stress coefficient [37, 38], computed as [16]

(5) Wε(x,t)=(1.2e(−0.35D))−0.2,D=0.611×[exp⁡(17.27×Ts−273Ts−36)    −exp⁡(17.27×Td−273Td−36)],

where *T*_*d*_ is dew point temperature (K) and *T*_*s*_ is surface temperature (K). When *T*_*s*_ − *T*_*d*_ < 0, *D* = 0. *T*_*d*_ was derived from Guo Jie's regression model for Sichuan province based on Yang Jingmei's findings of a significant linear relationship between dew point temperature and the logarithm of total perceptible water [39, 40] as follows:

(6) Ln(U)=1.8084+0.0735Td,

where *U* is total perceptible water (mm).

### 2.3. Validation of CASA

Generally, validation based on in situ data is relatively convincing. In this study, the in situ data of the three representative types of grasslands (alpine meadow, alpine shrub meadow, and mountain meadow) were compared with the results of the CASA model to conduct the validation. Alpine meadow, the largest grassland in Sichuan that covers half of the total grassland area, had 100 validation points, whereas the second largest grassland, alpine shrub meadow, had 30 validation points. Mountain meadow, with a smaller area, had 20 validation points. The CASA modeled NPP was extracted, which geographically and temporally corresponded to each in situ measured data point. The in situ measured data are actual dry yield (g/m^2^), which measures aboveground components, whereas the model results are NPP (gC/m^2^), including both above- and belowground parts. Therefore, conversions of in situ data were performed. The actual dry yield (g) multiplied by 0.45 was converted to the amount of carbon (gC) aboveground, and the root: shoot ratio was used to obtain the belowground allocation (see Table 1). As for the limitation of data acquisition, the ratio used here was derived from previously published literature.

The precision of the CASA modeled NPP was calculated as follows:

(7) Error=∑(|X1−X2|/X2)N×100%,

where *X*_1_ was the CASA modeled NPP, *X*_2_ was the amount of carbon converted from in situ measured data, and *N* was the number of validation points. The CASA model simulated the NPP of alpine meadow best with the highest precision of 76.2%, whereas mountain meadow had the lowest precision of 56.5%. The total precision for Sichuan grasslands was over 70% (see Table 2).

**Figure 1:**
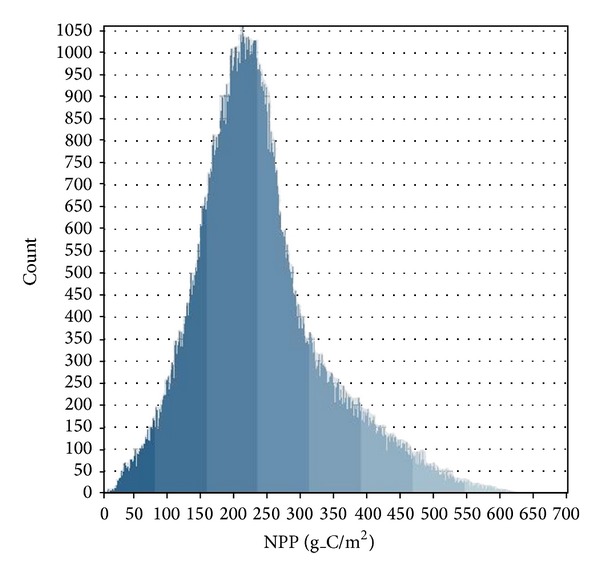
Histogram of Sichuan grassland NPP.

In addition to the validation based on in situ data, the modeled NPP was compared with published data. Histogram analysis of the modeled NPP was conducted (Figure 1), and the values of modeled NPP were between 150 and 250 gC/m^2^ which was consistent with Siyao et al.'s research [41].

### 2.4. Data Acquisition and Processing

#### 2.4.1. MODIS Data

The MODIS/Terra 8-day 1 km FPAR products MOD15A2, 8-day 1 km LST products MOD11A2, and diurnal 1 km total perceptible water products MOD05 for the Sichuan province in 2011 were acquired from the NASA website (http://reverb.echo.nasa.gov/). For MOD15A2 and MOD11A2, MRT (MODIS reprojection tools) was used for format and projection conversion and mosaic; for MOD05, HEG (HDF-EOS TO GEOTIFCONVERTION TOOL) was used. The preprocessed FPAR and LST data were grouped by month, and the monthly mean FPAR and LST were calculated. The missing value was handled with spline interpolation.

#### 2.4.2. Meteorological Data

The total solar surface radiation used in (2) was acquired from the Data Center for Resources and Environmental Sciences, Chinese Academy of Science, calculated using ANUSPLIN software [42].

**Figure 2:**
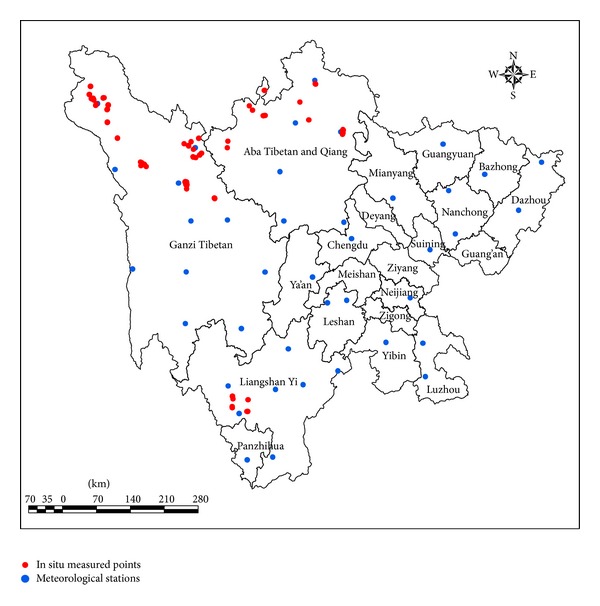
Distribution of in situ measurement locations and meteorological stations in Sichuan province.

The monthly mean temperature for Sichuan province in 2011 was derived from 41 meteorological stations (see Figure 2), acquired from the website of the China Meteorological Data Sharing Service System (http://cdc.cma.gov.cn/). The data included information on monthly mean temperature, elevation, latitude, and longitude, which was used to convert to grid monthly mean temperature using the method of spline interpolation. Elevation was taken into consideration in the interpolation as temperature decreased when elevation increased.

#### 2.4.3. In Situ Measurements

The in situ data were used to validate the model results, and they were derived from the grassland resource survey of the Sichuan province in 2011, which was conducted by the Sichuan Grassland General Work Station, China. There are 150 sample points of the three representative types of grasslands: alpine meadow (100), alpine shrub meadow (30), and mountain meadow (20) (see Figure 2). The sample points include grassland type, actual dry yield (g/m^2^), selection date, longitude, and latitude.

## 3. Results

### 3.1. Spatiotemporal Analysis of APAR for Sichuan Province

**Figure 3:**
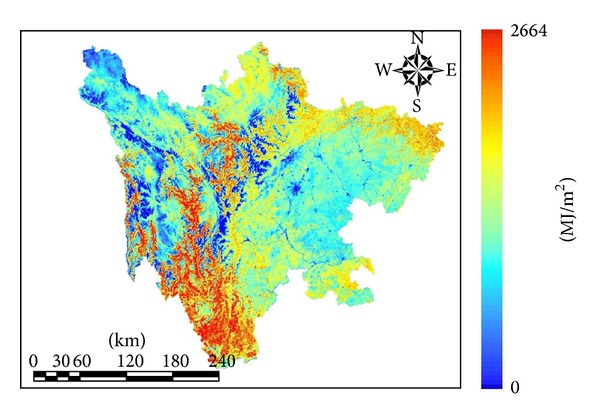
Distribution of APAR for Sichuan province in 2011.

The APAR of Sichuan province in 2011 (displayed in Figure 3) ranged from 0 to 2664.36 MJ/m^2^, with an average of 992.38 MJ/m^2^, totaling 480.7 × 10^12^ MJ/m^2^. The regions with higher values were located in southwest Sichuan province, which were mountainous locations with an elevation of 1000–3500 m and a subtropical climate. The higher APAR in this location might be attributed to the distribution of evergreen broad-leaf forest, where photosynthesis absorbs more solar radiation.

According to Table 3, APAR had a higher maximum (2569.33 MJ/m^2^ and 2664.36 MJ/m^2^) and average value (above 1200 MJ/m^2^) in the region of 2000–4000 m. This was because the evergreen broad-leaf forest area is concentrated in the region of 2600–4000 m, thus explaining the higher value of APAR. In the region of 4000–5000 m, APAR had a higher maximum of 2595.11 MJ/m^2^, but a lower average value of 678.07 MJ/m^2^. This result might be due to the reason of the area dominated by the grassland, which absorbed less solar radiation and determined the lower average, whereas less area of forest contributed to the higher maximum. Regions of elevation less than 1000 m were mainly plain and hilly regions, accounting for 29% of the province, with abundant rainfall and fertile soil. Approximately 27% of the APAR was concentrated in this area, as 70% of cultivated fields in Sichuan province were distributed in this location.

**Figure 4:**
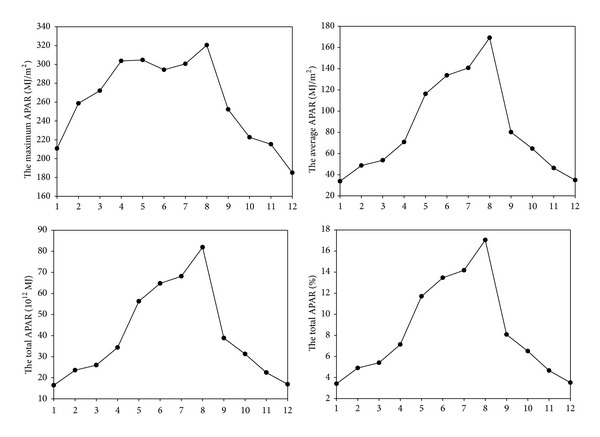
Monthly variation of APAR in 2011.

The APAR for Sichuan province in 2011 was measured monthly, and the trend of monthly variation throughout the year was represented with a broken line graph displayed in Figure 4. The first three months of the year had lower APAR. APAR increased in April and sharply increased in May. The APAR peaked in August after increasing monthly since June and then decreased. This might be attributed to the grassland beginning to turn green in April and generally turning green in May, whereas the main growing season is from June to August, and August was usually the most productive month of growth.

### 3.2. Spatiotemporal Analysis of LUE for Sichuan Grassland

**Figure 5:**
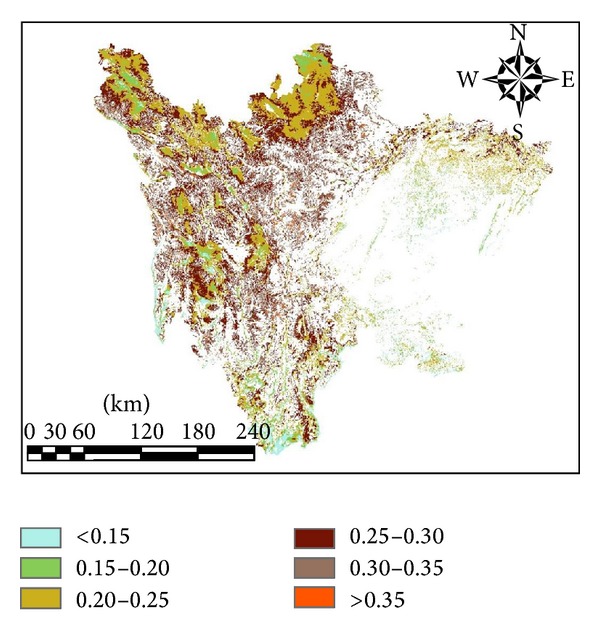
Distribution of LUE for Sichuan grassland in 2011.

The spatial distribution of Sichuan in 2011 was showed in Figure 5. Histogram analysis was conducted for Sichuan grassland, and the LUE ranged from 0.048 g/MJ to 0.514 g/MJ, concentrating at 0.2-0.3 g/MJ, with an average of 0.253 g/MJ. LUE partition statistics were conducted, and the results are shown in Table 4. In total, 36% of the LUE ranged from 0.25 g/MJ to 0.3 g/MJ, whereas 33% of the LUE ranged from 0.2 g/MJ to 0.25 g/MJ. Approximately, 10% of the LUE was less than 0.2 g/MJ or greater than 0.35 g/MJ. When the LUE increased, the average elevation had an increasing trend.

The average LUE for different types of Sichuan grassland is displayed in Table 5. LUE varied by grassland type, but the variation was not particularly obvious. Among the types mentioned in Table 5, the LUE for alpine shrub meadow was the highest, whereas the LUE for mountain woodland grass was the lowest.

**Figure 6:**
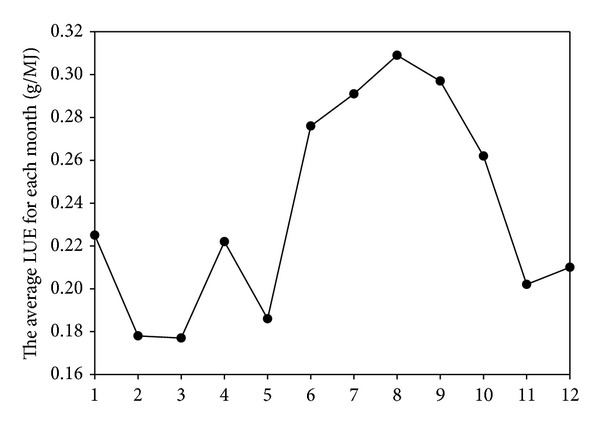
Monthly variation of LUE in 2011.

The LUE for Sichuan province in 2011 was measured monthly, and the monthly variation is displayed in Figure 6. The LUE had a lower value in autumn and winter and had a higher value in summer, with a peak in August. As shown in the figure, there was another peak in April, which may be attributed to the grassland turning green in April. In addition, environmental conditions such as rainfall and temperature were suitable for vegetation growth.

### 3.3. Spatiotemporal Analysis of NPP for Sichuan Grassland

**Figure 7:**
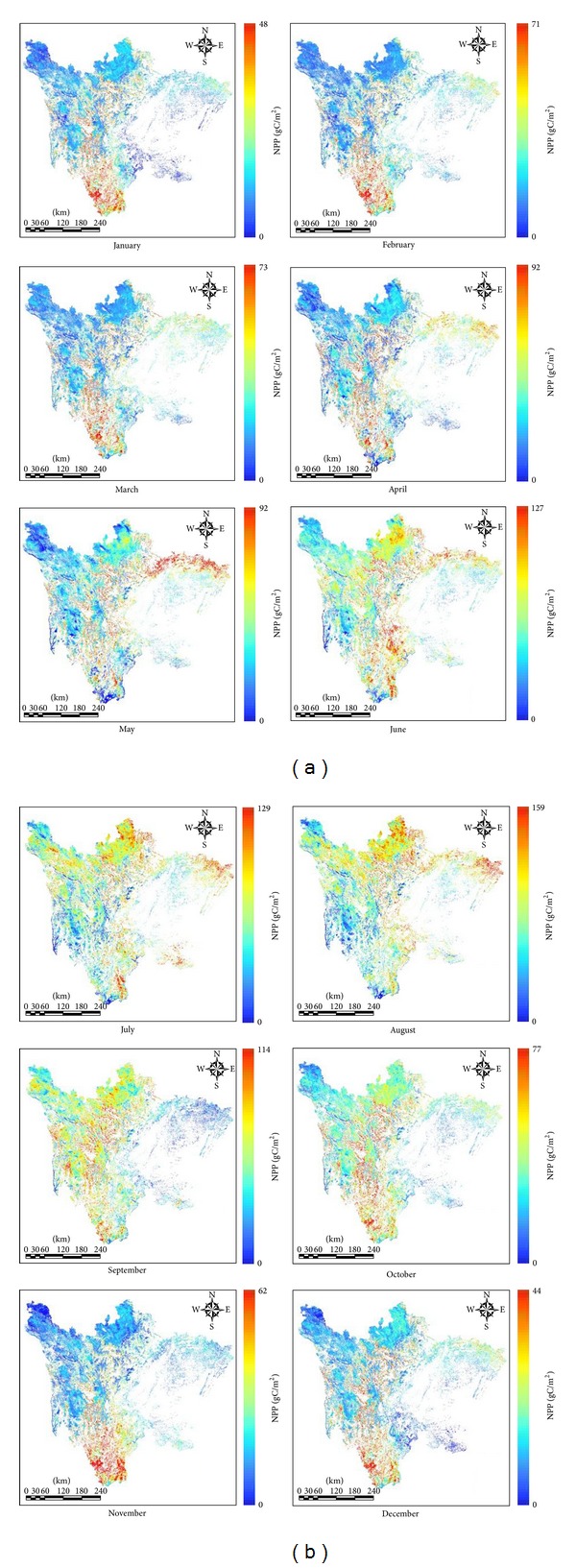
Distribution of NPP for Sichuan grassland for each month of 2011.

**Figure 8:**
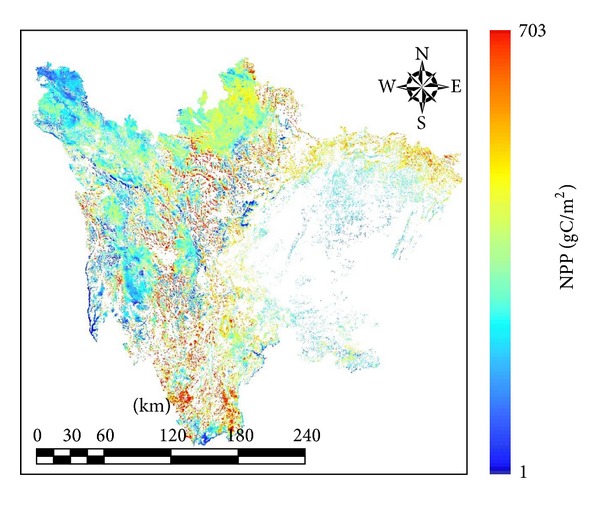
Distribution of NPP for Sichuan grassland in 2011.

Figure 7 shows the distribution of the NPP for Sichuan grassland areas for each month of 2011. From January to March, higher values of NPP were distributed in southwest Sichuan province, and the NPP in the northwest region was lower. The region of higher NPP gradually moved from the south to the north of Sichuan province after April. In May, the region of higher NPP appeared in northeast Sichuan province near the Sichuan Basin. During the growing season (June to August), the NPP in the northwest was higher. In September, the region of higher NPP transferred to the south, and a higher NPP appeared again in the southwest. The southwest area of Sichuan province, with a lower elevation, was dominated by mountain meadow and mountain woodland grass, whereas the northwest, with a higher elevation, was dominated by alpine meadow. Overall, the NPP in the southwest was slightly higher than that of the northwest (Figure 8).

Partition statistics based on elevation were performed, and the results are shown in Table 6. The region of 3000–4000 m included nearly 30% of grassland and 33% of the NPP, with an average NPP of 296.81 gC/m^2^. The region of 4000–5000 m included more than 40% of the grassland area and approximately 35% of the NPP, with the average NPP of 179.23 gC/m^2^. In the less than 2000 m and more than 5000 m elevations, neither the area nor NPP was less than 20%. Therefore, Sichuan grassland was concentrated in the region of 3000–5000 m, and there was little grassland distributed in elevations less than 2000 m or more than 5000 m. The productivity of grassland at 3000–4000 m elevation was higher than that of 4000–5000 m.

The most widely distributed grassland was alpine meadow, accounting for 50% of the NPP. Alpine shrub meadow followed, with the area and NPP accounting for approximately 15%. Subalpine woodland meadow and mountain grassland had higher average NPPs at 296.88 TgC and 288.98 TgC, respectively. Alpine meadow and alpine marsh grassland had lower average NPP values at 209.11 TgC and 223.92 TgC, respectively (see Table 7).

Partition statistics based on administrative distinction were performed; results were shown in Table 8. Ganzi Tibetan Autonomous Prefecture had the highest NPP, accounting for nearly 40%, and Aba Tibetan and Qiang Autonomous Prefecture followed, accounting for 26.7%. Liangshan Yi Autonomous Prefecture was the third highest region with about 15%. The three administrative regions mentioned above concentrated more than 80% of Sichuan grassland.

**Figure 9:**
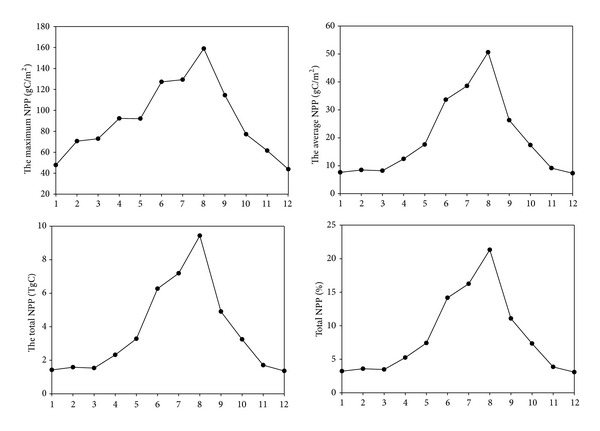
Monthly variation of NPP in 2011.

NPP for Sichuan province of 2011 was conducted monthly statistics, and the monthly variation was displayed in Figure 9. The accumulation of NPP was gradually increased since April and peaked in August. It might be attributed to the fact that Sichuan grassland began to turn green in April and vigorously grew in August. The accumulation of NPP from May to September, the main growing season, was up to 75% of the total.

## 4. Conclusions and Discussion

The MODIS data driven CASA model was used to simulate NPP of Sichuan grassland 2011, and the main conclusions of the study were as follows.

1. The overall precision reached 70%, while alpine meadow had the best precision of more than 75% among all three types of grassland validated.
2. Region of higher APAR was the southwest of the province with the elevation of 2000–4000 m, and APAR of 2011 peaked in August.
3. LUE varied among different types of grassland. Alpine shrub meadow had the highest LUE, while mountain woodland grass had the lowest. LUE of 2011 peaked in April and August, respectively.
4. Sichuan grassland NPP was concentrated in the region of 3000–5000 m. The most widely distributed type of grassland was alpine meadow, NPP of which in 2011 accounted for 50% of the total grassland. More than 80% of NPP distributed in Ganzi Tibetan Autonomous Prefecture, Aba Tibetan, and Qiang Autonomous Prefecture and Liangshan Yi Autonomous Prefecture. NPP peaked in August, and the accumulation of growing season was up to 75% of total amount of the year.

Photosynthesis is a complex process of physiology and ecology and the simulation is a challenging task. To reduce uncertainties during the process of establishing the CASA model, several steps have been performed. Firstly, the key parameters of the CASA model were determined with intensive review of existing literatures, especially cases conducted in the similar regions. Secondly, in situ observations from 150 points were adopted to evaluate the results of simulation. Finally, besides NPP, spatiotemporal analysis for APAR and LUE was also conducted. Those findings may present benefits for further researches. After that, uncertainty involved in the study may be discussed as follows. The CASA model simplified process based on photosynthetically active radiation absorbed by vegetation and light use efficiency and parameters of the model may affect the precision of simulation. In this study, the monthly mean temperature was calculated by spline function interpolation. Although the interpolation, taking elevation into consideration, generally reflected the temperature changes of the main topography, there were limitations on accurate expression of how local region temperature affected vegetation productivity for complex topography of Sichuan province. In addition, the actual dry yield was converted to NPP by empirical coefficient such as root: shoot ratio, which was used to validate the modeled NPP. The empirical coefficients were a source of error and spatial heterogeneity of pixels might be contributed to the uncertainties.

## Figures and Tables

**Table 1:** Root : shoot ratio of the three representative grassland types.

Grassland type	Ratio	Reference
Mountain meadow	6.23	Shilong Piao and Guo [32]
Alpine meadow	7.92	Li and Zhou (1998) [33]
Alpine shrub meadow	7.92	Li and Zhou (1998) [33]

**Table 2:** Validation results.

Grassland type	Validation points	Error	Precision
Alpine meadow	100	23.8%	76.2%
Alpine shrub meadow	30	33.4%	66.6%
Mountain meadow	20	43.5%	56.5%

Total	150	28.3%	71.7%

**Table 3:** Distribution of APAR for different ranges of elevation for Sichuan province in 2011.

Range of elevation (m)	Area (km^2^)	Percentage of area (%)	The highest APAR (MJ/m^2^)	The average APAR (MJ/m^2^)	The total APAR (10^12^ MJ)	Percentage of the total APAR (%)
<1000	138975	28.69	1951.38	966.59	128.74	26.78
1000–2000	51457	10.62	2662.98	1172.23	60.04	12.49
2000–3000	55056	11.37	2569.33	1238.93	68.14	14.18
3000–4000	104291	21.53	2664.36	1211.74	123.81	25.76
4000–5000	130678	26.98	2595.11	678.07	98.43	20.48
>5000	3917	0.81	2277.45	237.49	1.52	0.32

**Table 4:** LUE partition statistics of Sichuan grassland in 2011.

LUE (g/MJ)	Area (km^2^)	Area percentage (%)	Average elevation (m)
<0.15	5414	2.90	1741.76
0.15–0.2	19397	10.41	2173.04
0.2–0.25	61849	33.18	3107.58
0.25–0.3	67633	36.28	3776.54
0.3–0.35	29298	15.72	4044.05
>0.35	2806	1.51	4560.19

**Table 5:** Average LUE for different types of Sichuan grassland in 2011.

Grassland type	Average LUE (g/MJ)
Alpine meadow	0.261
Alpine marsh grass	0.235
Alpine shrub meadow	0.276
Subalpine woodland meadow	0.266
Mountain meadow	0.254
Mountain woodland grass	0.214
Mountain shrub grass	0.229
Mountain grass	0.220

**Table 6:** NPP for different ranges of elevation.

Elevation range (m)	Area (km^2^)	Area percentage (%)	Average NPP (gC/m^2^)	Total NPP (TgC)	Total NPP percentage (%)
<1000	15825	8.49	204.79	3.29	7.44
1000–2000	17814	9.56	264.06	4.69	10.60
2000–3000	17952	9.63	307.83	5.52	12.47
3000–4000	53128	28.50	296.81	14.98	33.86
4000–5000	80147	43.00	179.23	15.58	35.21
>5000	1531	0.82	108.46	0.19	0.42

**Table 7:** Statistics of NPP for different types of Sichuan grassland in 2011.

Grassland type	Area (km^2^)	Area percentage (%)	Average NPP (TgC)	Total NPP (TgC)	Total NPP percentage (%)
Alpine meadow	99470	53.35	223.92	22.27	50.26
Alpine marsh grass	7562	4.06	209.11	1.58	3.57
Alpine shrub meadow	26772	14.36	250.19	6.70	15.11
Subalpine woodland meadow	3115	1.67	296.88	0.92	2.09
Mountain meadow	2954	1.58	288.98	0.85	1.93
Mountain woodland grass	16771	8.99	258.80	4.34	9.79
Mountain shrub grass	19752	10.59	264.78	5.23	11.80
Mountain grass	5163	2.77	242.44	1.25	2.82
Others	4895	2.63	221.35	1.16	2.63

**Table 8:** The region statistics of NPP for Sichuan grassland in 2011.

Region	Modeled NPP (TgC)	Percentage (%)
Ganzi Tibetan Autonomous Prefecture	17.49	39.52
Aba Tibetan and Qiang Autonomous Prefecture	11.82	26.70
Liangshan Yi Autonomous Prefecture	6.76	15.28
Others	8.18	18.50

All	44.25	100.00
